# Breast Cancer Awareness and Knowledge Among Women at a Municipal Hospital in Ghana: A Cross‐Sectional Study

**DOI:** 10.1002/cnr2.70347

**Published:** 2025-09-08

**Authors:** Richard Ofosuhene, Alfred Effah, Wilfred Sam‐Awortwi, Richmond Adu Boahen Boamah, Patricia Akosah, Christian Obirikorang

**Affiliations:** ^1^ School of Anesthesia, Komfo Anokye Teaching Hospital Kumasi Ghana; ^2^ Department of Molecular Medicine Kwame Nkrumah University of Science and Technology Kumasi Ghana; ^3^ St. Joseph's College of Education Bechem Ghana

**Keywords:** awareness, breast cancer, breast self‐examination, knowledge

## Abstract

**Background:**

Breast cancer is the most common cancer in women worldwide; early detection improves prognosis while reducing mortality and morbidity.

**Aims:**

This study evaluates awareness, knowledge, and health‐seeking behaviors related to breast cancer among women attending Bibiani Municipal Hospital in Ghana, where data on awareness is scarce.

**Methods and Results:**

This cross‐sectional study involved 160 women attending the Bibiani Municipal Hospital. Validated questionnaires were used to collect data on sociodemographic characteristics, awareness and knowledge of breast cancer, breast self‐examination (BSE), and health‐seeking behaviors. Categorical variables were presented as frequency and percentages. Logistic regression was used to determine the independent predictors of adequate knowledge of breast cancer. Statistical analysis was performed using SPSS (version 26.0) and GraphPad Prism (version 8.0). *p* < 0.05 was considered statistically significant. Most participants were within 36–50 years (47.5%) and had no formal education (30.6%). Only 14.4% reported a family history of breast cancer. The majority (87.5%) were aware of breast cancer. However, only about 44% and 46% exhibited adequate knowledge regarding the risk factors, signs, and symptoms of breast cancer. The majority identified obesity (87.5%) and family history (80.6%) of breast cancer as risk factors, while most participants also identified a lump (68.1%) and pain (60%) in the breast as signs and symptoms. Education, employment status, age, and awareness of breast cancer were significantly associated with knowledge of breast cancer (*p* < 0.05). Only 47.5% were familiar with BSE, and BSE was performed by just 35.5% of participants. The majority (74.4%) indicated they would seek immediate help for a breast lump.

**Conclusion:**

Despite high awareness of breast cancer, knowledge of its risk factors and the signs and symptoms was lacking, with over half unaware of breast self‐examination (BSE). Health education campaigns by women‐friendly organizations are crucial to improving awareness of symptoms, risk factors, and BSE in the Bibiani municipality.

## 1 Introduction

Breast cancer is the most common cancer among women globally, with 2.3 million new cases and 685 000 deaths reported in 2020 [1]. Incidence rates vary significantly by region, with the highest rates in Western Europe and North America, and the lowest in Africa and Asia [2]. Despite its lower incidence rates, breast cancer remains the leading cause of death among women in Africa, with the highest mortality rate of 17.3 per 100 000 compared to other regions of the world [3]. Sub‐Saharan African women face particularly high mortality rates, with an estimated 85 787 deaths out of 186 598 cases in low‐income countries [4]. In Ghana, breast cancer remains a major public health challenge, with approximately 2900 new cases annually and one‐eighth resulting in death [5, 6]. It is the leading cause of cancer‐related deaths among Ghanaian women, accounting for 16% of all cancers [5].

Several risk factors have been attributed to breast cancer. However, female sex constitutes one of the major factors associated with an increased risk of breast cancer, primarily because of the enhanced hormonal stimulation. Unlike men, who present insignificant estrogen levels, women have breast cells that are very vulnerable to this hormone [7]. Older age is also a well‐known risk factor for breast cancer, with the risk of developing breast cancer increasing as follows: the 1.5% risk at age 40, 3% at age 50, and more than 4% at age 70 [7, 8]. Other risk factors associated with breast cancer include family history, race/ethnicity, alcohol intake, smoking, and body mass index (BMI) [9, 10, 11].

Besides the more aggressive form of breast cancer in African women, the increased mortality is linked to insufficient public awareness, limited screening programs, and consequent late‐stage diagnoses, often after metastasis [12]. Early diagnosis of breast cancer via screening methods has been demonstrated to enhance prognosis while decreasing morbidity and mortality rates [13]. Breast cancer screening involves three modalities which include mammography, clinical breast examination (CBE) by trained professionals, and self‐breast examination (BSE) [14]. In the absence of structured screening programs, early detection of breast cancer is often reliant on women expressing concerns when they present symptoms, during routine healthcare visits, or through CBE. Failure to practice BSE due to unfamiliarity with the technique or its benefits, avoidance of CBE, or foregoing mammography screening increases the likelihood of women being diagnosed with advanced‐stage breast cancer compared to those who adhere to screening recommendations [14].

Studies indicate that women's knowledge and beliefs regarding breast cancer significantly influence their tendencies to seek medical assistance [15]. Breast cancer often presents as a painless lump, but other symptoms can occur. Early hospital presentation depends on women recognizing symptoms and being informed about breast health. When women possess accurate information about breast cancer, including risk factors, signs, and symptoms, they are more likely to recognize potential abnormalities and seek medical assistance promptly. Conversely, insufficient awareness may lead to delayed diagnosis and treatment, exacerbating the severity of the disease and reducing survival rates.

Despite the importance of early diagnosis of breast cancer, there remains limited data regarding the awareness of breast cancer risk factors and screening among women within the Bibiani Municipality, Ghana. The Bibiani Municipality is a region with limited data on breast cancer awareness, and women in this area may face unique barriers to early detection, such as limited access to healthcare services, cultural beliefs, and health literacy challenges. Assessing women's knowledge of breast cancer and BSE practice in this population is essential for enhancing early detection efforts and guiding targeted public health interventions. The Bibiani Municipal Hospital serves as a major healthcare provider in the municipality, offering medical services to women from diverse socioeconomic backgrounds. This study aimed to evaluate the level of awareness, knowledge, and health‐seeking behaviors pertaining to breast cancer, along with the factors associated with them, among women attending the Bibiani Municipal Hospital in Ghana. Findings from this study could help inform policies and educational programs to enhance breast cancer awareness and screening uptake in this area.

## 2 Materials and Methods

### 2.1 Study Design

This study employed a cross‐sectional study design to recruit participants at the Bibiani Government Hospital, from January to August 2024. A cross‐sectional study was employed because it is relatively inexpensive and can be completed over a short period.

### 2.2 Profile of Study Area

Bibiani is a municipality located in the western north region of Ghana. It serves as an important healthcare hub, providing medical services to the local population and surrounding communities. The area is characterized by a mix of urban and rural settings, with diverse socioeconomic backgrounds and cultural practices. The hospital serves as a referral center in the western north region, part of Ahafo region, Ashanti region, Western region, and Central region. The hospital is equipped with modern facilities for early diagnosis and screening services.

### 2.3 Study Population

The study population comprised women who attended the out‐patient department at the Bibiani Municipal Hospital. Those who met the study's inclusion criteria were enrolled in the study.

### 2.4 Sampling Size and Procedure

The sample size was determined using Slovin's formula.

n=N1+Ne^2

where *n* is the sample size, *N* is the population size is the women who access healthcare in the hospital during the study period = 150 and *e* is the confidence level/margin of error (0.05).

n=1501+1500.05^2=109.1

The minimum sample required for the study was 109; however, a total of 160 participants were recruited to improve statistical power.

### 2.5 Sampling Technique

A simple random sampling technique was used in selecting the participants for the study. At the outpatient department, participants were asked to randomly pick cards marked with either “0” or “1.” Those who picked a card marked “1” were included in the study, while those who picked “0” were excluded. This sampling method was chosen because it ensures that all respondents have equal chances of being selected for the study, with no subjective bias on the part of the researcher.

### 2.6 Inclusion and Exclusion Criteria

The study enrolled women aged 18 years and above who attended the Bibiani Government Hospital during the time of study, after voluntarily consenting to participate. However, women with mental incapacities were excluded, as they may have difficulty accurately recalling past events, which could affect the reliability of their responses.

### 2.7 Data Collection Tools

A well‐structured questionnaire was used to collect data from study participants. It was divided into four sections (A–D). Part A included questions on the general characteristics of participants, such as sociodemographic data and family history of breast cancer. Parts B to D assessed participants' knowledge and awareness of breast cancer and BSE. The questionnaire included questions adapted from previously published studies that had used validated instruments [5, 16]. A total of 12 questions, based on dichotomous “Yes” or “No” answers, were used to assess participants' knowledge of the risk factors for breast cancer. A correct answer was given a score of 1, while a wrong answer received a score of 0, resulting in a maximum score of 12. A total score of 6 or more was classified as “adequate knowledge,” while a score below 6 was classified as “inadequate knowledge.” Similarly, knowledge of the signs and symptoms of breast cancer was assessed using 5 dichotomous “Yes” or “No” questions. A correct answer was scored 1, and a wrong answer was scored 0, leading to a maximum possible score of 5. A total score of 3 or more was classified as “adequate knowledge,” while a score below 3 was classified as “inadequate knowledge”.

### 2.8 Data Collection Procedure

Data for this study were obtained through face‐to‐face interviews using a well‐structured questionnaire. Trained research assistants read each item and documented participants' responses. The study's questionnaires were administered in either English or Ghanaian language (Twi), the predominant local language, with interviewers providing real‐time translation when necessary. All research assistants involved in the data collection process were proficient in both languages and received standardized training to ensure consistency. To maintain participant confidentiality, interviews were conducted in private locations, and no personally identifiable information (such as names or telephone numbers) was collected.

### 2.9 Data Handling and Analysis

The data collected were cleaned and coded in Microsoft Excel (2019). The Statistical Package for Social Sciences (SPSS, version 26.0) and GraphPad Prism (version 8.0) were used to perform the statistical analysis. Categorical variables were presented as frequencies and percentages (%). The χ^2^ test and logistic regression were used to determine the association between study variables and knowledge of breast cancer. *p* < 0.05 was considered statistically significant.

## 3 Results

### 3.1 Baseline Characteristics of Study Participants

Presented in Table 1 are the sociodemographic characteristics of study participants and their family history of breast cancer. Of the 160 participants enrolled in the study, the majority were aged between 36 and 50 years (47.5%), married (70.6%), and identified as Christian (68.1%). Considering educational background, approximately 27% had tertiary education, while a notable number had received no formal education (30.6%). Moreover, the majority of the study participants were self‐employed (44.4%), while 35% were unemployed. Most participants reported no family history of breast cancer (85.6%), and the majority had between 3 and 5 children (53.8%) (Table 1).

**TABLE 1:** Sociodemographic characteristics of study participants.

Variables	Frequency (*n* = 160)	Percentage (%)
Age group (years)		
18–25	26	16.3
26–35	58	36.3
36–50	76	47.5
Marital status		
Married	113	70.6
Single	37	23.1
Divorced/widowed	10	6.3
Religion		
Christian	109	68.1
Muslim	51	31.9
Education		
No formal education	49	30.6
Basic education	28	17.5
SHS	15	9.4
Tertiary	43	26.9
Technical/vocational	25	15.6
Employment status		
Unemployed	56	35.0
Self employed	71	44.4
Employed	33	20.6
Family history of breast cancer		
No	137	85.6
Yes	23	14.4
No. of children		
0–2	58	36.3
3–5	86	53.8
Above 5	16	10.0

*Note:* Data presented as frequency and percentages (%).

Abbreviation: No., number.

### 3.2 Awareness of Breast Cancer Risk Factors, Signs and Symptoms Among Study Participants

Most participants (87.5%) had heard and were aware of breast cancer. However, only 44.4% and 46.3% had adequate knowledge regarding the risk factors and signs and symptoms of breast cancer, respectively (Table 2).

**TABLE 2:** Awareness, knowledge of breast cancer risk factors, signs and symptoms.

Variable	Frequency (*n* = 160)	Percentage (%)
Ever heard about breast cancer		
No	20	12.5
Yes	140	87.5
Knowledge on risk factors of breast cancer		
Inadequate knowledge	89	55.6
Adequate knowledge	71	44.4
Knowledge on sign and symptoms of breast cancer		
Inadequate knowledge	86	53.8
Adequate knowledge	74	46.3

*Note:* Data presented as frequency and percentages (%).

### 3.3 Awareness and Knowledge of Risk Factors for Breast Cancer

**FIGURE 1:**
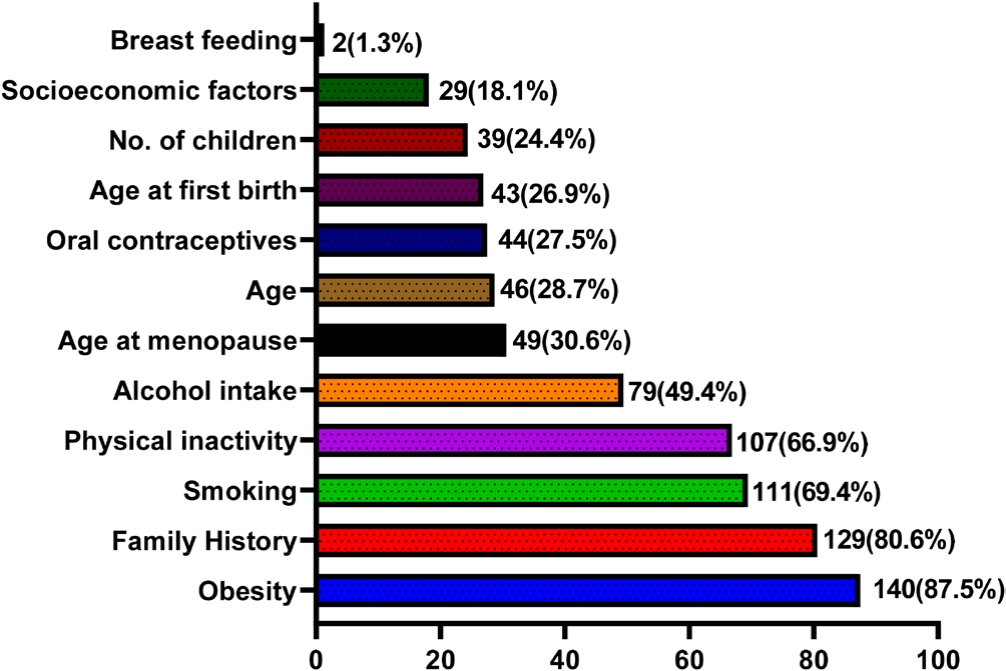
Percentage of correct answers regarding risk factors of breast cancer.

The risk factors most identified correctly were obesity (87.5%), family history (80.6%), smoking (69.4%) and physical inactivity (66.9%). Age (28.7%), use of oral contraceptives (27.5%), age at first birth (26.9%), number of children (24.4%), socioeconomic factors (18.1%) and breast feeding (1.3%) were the least identified risk factors of breast cancer (Figure 1).

### 3.4 Knowledge of Signs and Symptoms of Breast Cancer

**FIGURE 2:**
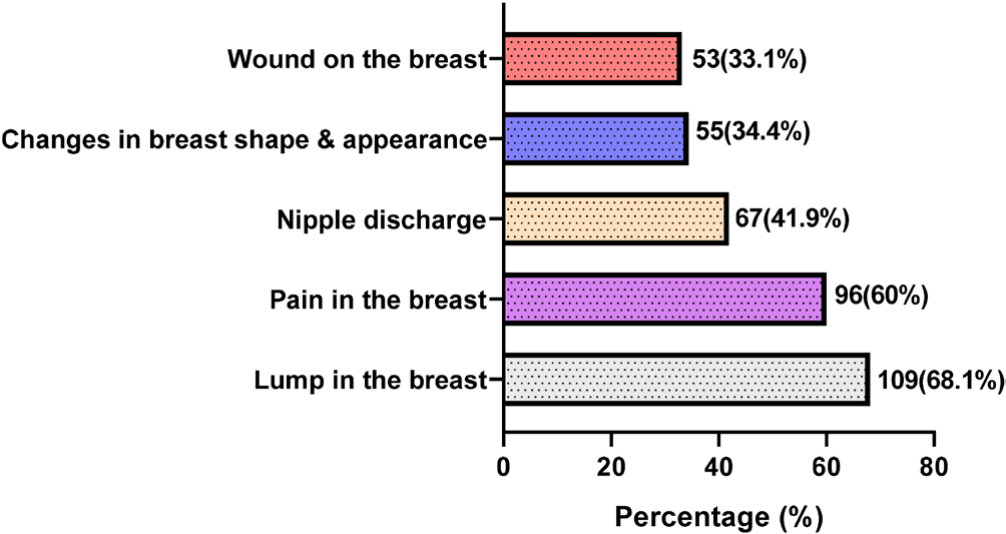
Percentage of correct answers regarding the signs and symptoms of breast cancer.

More than half of the study participants (53.8%) had inadequate knowledge regarding the signs and symptoms of breast cancer (Table 2). The signs and symptoms correctly identified were lump (68.1%) and pain in the breast (60%). Nipple discharge (41.9%), changes in breast shape and appearance (34.4%) and wound on the breast (33.1%) were the least identified signs and symptoms of breast cancer (Figure 2).

### 3.5 Sources of Breast Cancer Information Among Study Participants

**FIGURE 3:**
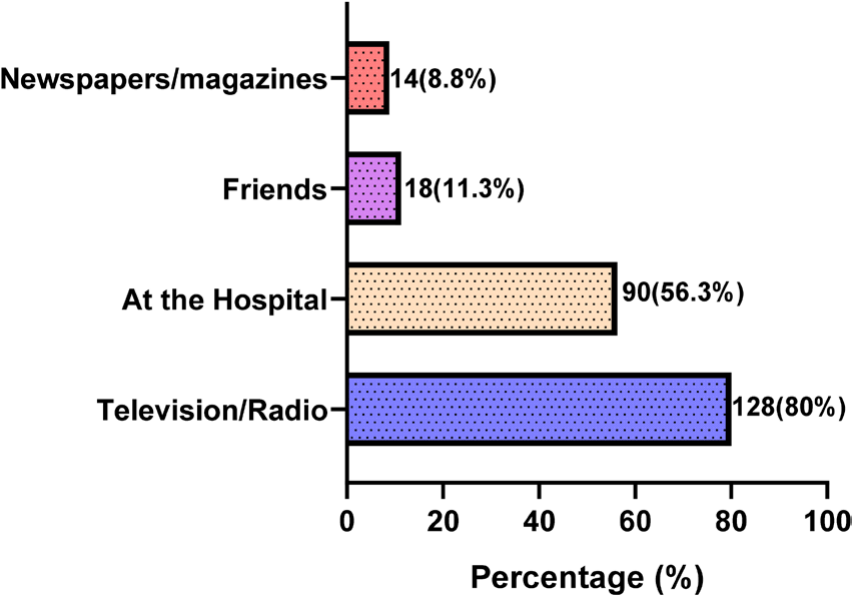
Sources of information about breast cancer.

The main source of information about breast cancer was the mass media such as television/radio (80%), followed by the hospital (56.3%), friends (11.3%) while few had information regarding breast cancer from newspapers/magazines (8.8%) (Figure 3).

### 3.6 Knowledge, Awareness and Breast Health Seeking Behavior Among Study Participants

Presented in Table 3 are the knowledge, awareness, and breast health‐seeking behavior among participants. Approximately 61% were aware of breast cancer screening, while 39% were not. About 42% of the study participants had clinically examined their breasts, with the remaining 58% reporting no prior clinical examination. Moreover, 47.5% of the participants knew about BSE, of which only 35.5% have ever performed BSE. The majority (74.4%) indicated they would seek immediate help for a breast lump, with smaller percentages opting for help within 1 week (11.3%) or 1 month (3.8%). Most participants (95.0%) stated they would visit a health facility if experiencing breast swelling, while very few would turn to faith healers (0.6%) or traditional healers (3.1%). Regarding the gender of healthcare providers, 93.8% were comfortable with male doctors examining their breasts, while fewer participants (33.8%) were willing to allow male traditional healers to do so. The decision of where to seek help for a breast problem was primarily made by the individuals themselves (83.8%), followed by husbands (11.9%) and parents (4.4%). Health centers (45.0%) and district hospitals (46.9%) were the preferred choices for seeking help, with regional hospitals being less frequently chosen (8.1%).

**TABLE 3:** knowledge, awareness, and breast health seeking behavior among study participants.

Variable	Frequency (*n* = 160)	Percentage (%)
Do you know about breast cancer screening?		
No	63	39.4
Yes	97	60.6
Have you clinically examined your breast before?		
No	93	58.1
Yes	67	41.9
Do you know about breast self‐examination (BSE)?		
No	84	52.5
Yes	76	47.5
Do you perform BSE? (*n* = 76)		
No	49	64.5
Yes	27	35.5
How soon would you seek help for a breast lump?		
Immediately	119	74.4
1 week	18	11.3
1 month	6	3.8
Depends on factors	17	10.6
Where would you go if you have a breast swelling?		
Health facility	152	95.0
Faith healer	1	0.6
Traditional healer	5	3.1
Don't know	2	1.3
Would you allow a male doctor to examine your breast?		
No	10	6.3
Yes	150	93.8
Would you allow a male traditional healer to examine your breast?		
No	106	66.3
Yes	54	33.8
Who decides where you would seek help for a breast problem?		
Myself	134	83.8
Husband	19	11.9
Parent	7	4.4
Which health facility would you go to seek help for a breast problem?		
Health center	72	45.0
District hospital	75	46.9
Regional hospital	13	8.1

*Note:* Data presented as frequency and percentages (%).

### 3.7 Sociodemographic Characteristics of Participants Associated With Knowledge on Risk Factors, Signs and Symptoms

Education level showed a notable association (*p* = 0.003), with individuals having tertiary education exhibiting higher levels of knowledge compared to those with no formal education or basic education. Moreover, employment status was significantly associated with knowledge (*p* = 0.001), revealing that unemployed participants were more likely to have inadequate knowledge compared to those who were self‐employed or employed (Table 4).

**TABLE 4:** Association between sociodemographic factors and knowledge on risk factors of breast cancer.

Variable	Knowledge on risk factors		*p*
	Inadequate knowledge (*n* = 89)	Adequate knowledge (*n* = 71)	
Age group (years)			0.659
18–25	14 (15.7)	12 (16.9)	
26–35	35 (39.3)	23 (32.4)	
36–50	40 (44.9)	36 (50.7)	
Marital status			0.574
Married	63 (70.8)	50 (70.4)	
Single	19 (21.3)	18 (25.4)	
Divorced/widowed	7 (7.9)	3 (4.2)	
Religion			0.829
Christian	60 (67.4)	49 (69.0)	
Muslim	29 (32.6)	22 (31.0)	
Education			**0.003**
No formal education	30 (33.7)	19 (26.8)	
Basic education	20 (22.5)	8 (11.3)	
SHS	9 (10.1)	6 (8.5)	
Tertiary	13 (14.6)	30 (42.3)	
Technical/vocational	17 (19.1)	8 (11.3)	
Employment status			**0.001**
Unemployed	28 (31.5)	28 (39.4)	
Self employed	50 (56.2)	21 (29.6)	
Employed	11 (12.4)	22 (31.0)	
Family history of breast cancer			0.719
No	77 (86.5)	60 (84.5)	
Yes	12 (13.5)	11 (15.5)	
No. of children			0.265
0–2	35 (39.3)	23 (32.4)	
3–5	48 (53.9)	38 (53.5)	
Above 5	6 (6.7)	10 (14.1)	
Ever heard of breast cancer			0.674
No	12 (13.5)	8 (11.3)	
Yes	77 (86.5)	63 (88.7)	

*Note:* Data presented as frequency (%), χ^2^ test *p*‐values were presented, *p* < 0.05 was considered statistically significant. Bolded *p*‐values were statistically significant.

Table 5 shows the factors associated with participants' knowledge of the signs and symptoms of breast cancer. The study revealed a statistically significant association between age and knowledge of the signs and symptoms of breast cancer (*p* = 0.043), with individuals aged 36–50 years (55.8%) having inadequate knowledge compared to the younger age groups. Education level was significantly associated with knowledge of breast cancer signs and symptoms (*p* < 0.001), with most individuals (42.3%) having tertiary education displaying adequate knowledge compared to those with lower educational attainment. Moreover, only a few of the participants with adequate knowledge were unemployed (31.1%) compared to those employed. There was a statistically significant association between knowledge regarding the signs and symptoms of breast cancer and employment status (*p* = 0.001), following a χ^2^ test analysis. Similarly, there was a significant association between prior awareness of breast cancer and knowledge of breast cancer signs and symptoms (*p* = 0.042), with over 93% of individuals with adequate knowledge having heard about breast cancer.

**TABLE 5:** Association between sociodemographic factors and knowledge on signs and symptoms of breast cancer.

Variable	Knowledge on signs and symptoms		*p*
	Inadequate knowledge (*n* = 86)	Adequate knowledge (*n* = 74)	
Age group (years)			**0.043**
18–25	14 (16.3)	12 (16.2)	
26–35	24 (27.9)	34 (45.9)	
36–50	48 (55.8)	28 (37.8)	
Marital status			0.900
Married	60 (69.8)	53 (71.6)	
Single	21 (24.4)	16 (21.6)	
Divorced/widowed	5 (5.8)	5 (6.8)	
Religion			0.888
Christian	59 (68.6)	50 (67.6)	
Muslim	27 (31.4)	24 (32.4)	
Education			**< 0.001**
No formal education	37 (43.0)	12 (16.2)	
Basic education	16 (18.6)	12 (16.2)	
SHS	11 (12.8)	4 (5.4)	
Tertiary	11 (12.8)	32 (43.2)	
Technical/vocational	11 (12.8)	14 (18.9)	
Employment status			**0.001**
Unemployed	33 (38.4)	23 (31.1)	
Self employed	45 (52.3)	26 (35.1)	
Employed	8 (9.3)	25 (33.8)	
Family history of breast cancer			0.129
No	77 (89.5)	60 (81.1)	
Yes	9 (10.5)	14 (18.9)	
No. of children			0.921
0–2	30 (34.9)	28 (37.8)	
3–5	47 (54.7)	39 (52.7)	
Above 5	9 (10.5)	7 (9.5)	
Ever heard of breast cancer			**0.042**
No	15 (17.4)	5 (6.8)	
Yes	71 (82.6)	69 (93.2)	

*Note:* Data presented as frequency (%), χ^2^ test *p*‐values were presented, *p* < 0.05 was considered statistically significant. Bolded *p*‐values were statistically significant.

However, marital status, religion, family history of breast cancer, and number of children were not associated with knowledge regarding the signs and symptoms of breast cancer (*p* > 0.05) (Table 5).

### 3.8 Predictors of Adequate Knowledge of Risk Factors Associated With Breast Cancer

In a univariate logistic regression analysis, having tertiary education [(cOR = 4.90, 95% CI (1.69–14.20), *p* = 0.003)] was significantly associated with higher odds of adequate knowledge of risk factors of breast cancer. However, compared to those employed, being self‐employed [(cOR = 0.21, 95% CI (0.09–0.51), *p* = 0.001)] was associated with a lower likelihood of having adequate knowledge regarding risk factors of breast cancer. Factors including age, marital status, religion, family history of breast cancer, and awareness of breast cancer did not show any significant association with knowledge (*p* > 0.05).

After adjusting for putative confounders in a multivariate logistics regression analysis, having tertiary education [(aOR = 5.31, 95% CI (1.25–22.56), *p* = 0.024)] and having children between 0 and 2 [(aOR = 0.09, 95% CI (0.02–0.47), *p* = 0.005)] or children between 3 and 5 [(aOR = 0.27, 95% CI (0.08–0.96), *p* = 0.044)] were the independent predictors of adequate knowledge of risk factors of breast cancer (Table 6).

**TABLE 6:** Univariate and multivariate logistic regression analysis, predicting adequate knowledge on risk factors of breast cancer.

Variable	Adequate knowledge (*n* = 71)	cOR (95% CI)	*p*	aOR (95% CI)	*p*
Age group (years)					
18–25	12 (16.9)	0.95 (0.39–2.33)	0.915	0.62 (0.13–3.00)	0.555
26–35	23 (32.4)	0.73 (0.37–1.46)	0.373	0.69 (0.28–1.69)	0.421
36–50 (Ref)	36 (50.7)	1.00	—	1.00	—
Marital status					
Married	50 (70.4)	1.85 (0.47–7.53)	0.389	1.15 (0.22–6.09)	0.873
Single	18 (25.4)	2.21 (0.49–9.89)	0.299	4.23 (0.57–31.41)	0.159
Divorced/widowed (Ref)	3 (4.2)	1.00	—	1.00	—
Religion					
Christian	49 (69.0)	1.08 (0.55–2.12)	0.829	0.85 (0.38–1.89)	0.693
Muslim (Ref)	22 (31.0)	1.00	—	1.00	—
Education					
No formal education	19 (26.8)	1.35 (0.49–3.73)	0.567	0.76 (0.23–2.47)	0.647
Basic education	8 (11.3)	0.85 (0.26–2.75)	0.786	0.80 (0.22–2.95)	0.740
SHS	6 (8.5)	1.42 (0.37–5.37)	0.608	1.38 (0.27–6.95)	0.699
Tertiary	30 (42.3)	4.90 (1.69–14.20)	**0.003**	5.31 (1.25–22.56)	**0.024**
Technical/vocational (Ref)	8 (11.3)	1.00	—	1.00	—
Employment status					
Unemployed	28 (39.4)	0.50 (0.21–1.22)	0.128	1.51 (0.38–6.08)	0.562
Self employed	21 (29.6)	0.21 (0.09–0.51)	**0.001**	0.58 (0.15–2.24)	0.426
Employed (Ref)	22 (31.0)	1.00	—	1.00	—
Family history of breast cancer					
No	60 (84.5)	0.85 (0.35–2.06)	0.719	0.60 (0.20–1.79)	0.362
Yes (Ref)	11 (15.5)	1.00	—	1.00	—
No. of children					
0–2	23 (32.4)	0.39 (0.13–1.23)	0.110	0.09 (0.02–0.47)	**0.005**
3–5	38 (53.5)	0.48 (0.16–1.42)	0.184	0.27 (0.08–0.96)	**0.044**
Above 5 (Ref)	10 (14.1)	1.00	—	1.00	—
Ever heard of breast cancer					
No	8 (11.3)	0.82 (0.31–2.12)	0.674	1.56 (0.50–4.83)	0.441
Yes (Ref)	63 (88.7)	1.00	—	1.00	—

*Note:* Univariate and multivariate logistic regression analyses *p*‐values are presented, *p* < 0.05 and bolded was considered statistically significant.

Abbreviations: aOR, adjusted odd ratios, CI, confidence interval; cOR, crude odd ratios.

### 3.9 Predictors of Adequate Knowledge of the Signs and Symptoms of Breast Cancer

In a univariate logistic regression analysis, compared to those aged 36–50 years, participants within 26–35 years [(cOR = 2.43, 95% CI (1.21–4.89), *p* = 0.013)] had higher odds of adequate knowledge of the signs and symptoms of breast cancer. However, compared to those employed, being unemployed [(cOR = 0.22, 95% CI (0.09–0.58), *p* = 0.002)], self‐employed [(cOR = 0.19, 95% CI (0.07–0.47), *p* < 0.001)], having no formal education [(cOR = 0.26, 95% CI (0.09–0.71), *p* = 0.009)] and being unaware of breast cancer [(cOR = 0.34, 95% CI (0.12–0.99), *p* = 0.049)] were associated with a lower likelihood of having adequate knowledge of the signs and symptoms of breast cancer. Factors including marital status, religion, and family history of breast cancer did not show any significant association with knowledge of the signs and symptoms of breast cancer (*p* > 0.05).

After adjusting for putative confounders in a multivariate logistics regression analysis, being within the age group of 36–50 years [(aOR = 3.44, 95% CI (1.34–8.85), *p* = 0.010)] and having no formal education [(aOR = 0.22, 95% CI (0.07–0.73), *p* = 0.014)] were the independent predictors of adequate knowledge on the signs and symptoms of breast cancer (Table 7).

**TABLE 7:** Univariate and multivariate logistic regression analysis, predicting adequate knowledge on signs and symptoms of breast cancer.

Variable	Adequate knowledge (*n* = 74)	cOR (95% CI)	*p*	aOR (95% CI)	*p*
Age group (years)					
18–25	12 (16.2)	1.47 (0.60–3.62)	0.402	4.07 (0.77–21.45)	0.098
26–35	34 (45.9)	2.43 (1.21–4.89)	**0.013**	3.44 (1.34–8.85)	**0.010**
36–50 (Ref)	28 (37.8)	1.00	—	1.00	—
Marital status					
Married	53 (71.6)	0.88 (0.24–3.22)	0.851	0.41 (0.08–2.20)	0.300
Single	16 (21.6)	0.76 (0.19–3.09)	0.703	0.25 (0.03–1.94)	0.184
Divorced/widowed (Ref)	5 (6.8)	1.00	—	1.00	—
Religion					
Christian	50 (67.6)	0.95 (0.49–1.86)	0.888	0.78 (0.35–1.72)	0.535
Muslim (Ref)	24 (32.4)	1.00	—	1.00	—
Education					
No formal education	12 (16.2)	0.26 (0.09–0.71)	**0.009**	0.22 (0.07–0.73)	**0.014**
Basic education	12 (16.2)	0.59 (0.20–1.75)	0.341	0.43 (0.12–1.48)	0.180
SHS	4 (5.4)	0.29 (0.07–1.15)	0.077	0.20 (0.04–1.05)	0.057
Tertiary	32 (43.2)	2.29 (0.80–6.50)	0.121	1.27 (0.30–5.42)	0.747
Technical/vocational (Ref)	14 (18.9)	1.00	—	1.00	—
Employment status					
Unemployed	23 (31.1)	0.22 (0.09–0.58)	**0.002**	0.55 (0.12–2.40)	0.422
Self employed	26 (35.1)	0.19 (0.07–0.47)	**< 0.001**	0.35 (0.08–1.52)	0.161
Employed (Ref)	25 (33.8)	1.00	—	1.00	—
Family history of breast cancer					
No	60 (81.1)	0.50 (0.20–1.23)	0.133	0.54 (0.17–1.65)	0.278
Yes (Ref)	14 (18.9)	1.00	—	1.00	—
No. of children					
0–2	28 (37.8)	1.20 (0.39–3.66)	0.748	0.28 (0.54–1.46)	0.131
3–5	39 (52.7)	1.07 (0.36–3.13)	0.906	0.30 (0.08–1.14)	0.076
Above 5 (Ref)	7 (9.5)	1.00	—	1.00	—
Ever heard of breast cancer					
No	5 (6.8)	0.34 (0.12–0.99)	**0.049**	0.57 (0.18–1.86)	0.570
Yes (Ref)	69 (93.2)	1.00	—	1.00	—

*Note:* Univariate and multivariate logistic regression analyses *p*‐values are presented, *p* < 0.05 and bolded was considered statistically significant.

Abbreviations: aOR, adjusted odd ratios; CI, confidence interval; cOR, crude odd ratios.

## 4 Discussion

The mortality rate of breast cancer is significantly high among Sub‐Saharan African women compared to those in Western nations, despite Western women having a higher incidence rate [3, 17]. This disparity is largely due to limited public awareness, inadequate screening programs, and late‐stage diagnoses [12]. Early detection through screening has been shown to improve prognosis and reduce mortality [13]. Previous studies have shown that women's knowledge and beliefs about breast cancer are associated with their likelihood of seeking medical help, and that practicing BSE is significantly linked to early detection [18]. Despite the importance of early diagnosis of breast cancer, there remains limited data regarding the awareness of breast cancer risk factors and screening among women within the Bibiani Municipality, Ghana. We evaluated the level of awareness, knowledge, and health‐seeking behaviors pertaining to breast cancer, along with the factors associated with them, among women attending the Bibiani Municipal Hospital in Ghana. In this study, most of the participants (87.5%) were aware of breast cancer. However, only approximately 44% and 46% exhibited adequate knowledge regarding the risk factors, signs, and symptoms of breast cancer, respectively.

The proportion of awareness (87.5%) observed in this study was slightly higher than that reported by a previous study in Ghana [5], but consistent with the 88% reported among women at the Makola market in Accra, Ghana [19]. Similarly, this finding concurs with the 84% previously reported among reproductive‐aged women in Ghana [20], and 88.1% among college students in Cameroon [21]. Most of these previous studies were conducted among students; however, our study included women from the general population, potentially explaining the slight variations in awareness levels observed. Student organizations often conduct annual programs focusing on female health issues like cervical and breast cancers, boosting awareness among students. Higher awareness typically correlates with increased recognition of the importance of regular screenings and active participation in screening programs. This heightened engagement is crucial for detecting breast cancer early, when treatment is most effective.

The primary source of information about breast cancer was mass media, specifically television and radio. Consistently, other studies have also identified television and radio as the predominant sources of information about breast cancer [16, 22]. The significant breast cancer awareness observed among participants in the study may be credited to the higher number of private television and radio stations in the country. These stations often broadcast programs in local languages, appealing to a wide audience and contributing to increased awareness levels. Given that the media, particularly radio and television, were the two most prominent sources of information on the condition, they must be utilized by taking advantage of popular programs to have slots for discussing matters related to breast cancer. Targeted advertising campaigns and educational programs on radio and television could be developed for specific demographic groups, particularly in rural or low‐income areas, to increase awareness of breast cancer, BSE, and available screenings.

Understanding of breast cancer risk factors and signs and symptoms was relatively low, with only approximately 44% and 46% of participants demonstrating adequate knowledge, respectively. More than half of the participants failed to identify age, alcohol consumption, use of oral contraceptives, and age at menopause as risk factors for breast cancer, along with symptoms such as wounds on the breast, nipple discharge, and changes in shape and appearance as signs of the disease. A similar lack of knowledge was reported among Ghanaian health professionals [5]. A study by Al‐Mousa and colleagues among females in Jordan found that only 53.7% of participants had an intermediate level of knowledge regarding breast cancer risk factors, while 44% were rated as having good to excellent knowledge about breast cancer signs and symptoms [13]. Our findings also reflect reports from prior research in Ethiopia, Nigeria, and Egypt, where understanding of risk factors for breast cancer was found to be poor [23, 24, 25]. The current knowledge gaps may influence people's health‐seeking behaviors and willingness to undergo breast screening, which might result in delayed diagnosis, which could lead to complications and even death. Therefore, it is imperative for stakeholders to put in place measures to raise awareness of breast cancer risk factors and clinical presentation.

While most study participants were aware of breast cancer screening and had prior experience with clinical breast examinations, only a small percentage (47.5%) were familiar with BSE, and among them, just 35.5% had ever performed BSE. This finding confirms the reports from a previous study in the Volta region of Ghana, which revealed that in spite of the high awareness (88.3%) of breast cancer, only 27.5% practiced BSE [26]. A similar study in Cameroon reported that although 50% of women had heard of BSE, only 34.8% practiced it, and among those, 71.6% demonstrated poor technique or irregular practice [27]. Similarly, another study by Conte et al. reported a lack of BSE practice among Italian women [28]. However, these results are inconsistent with a previous study conducted in Ghana, which reported that over 90% of participants were aware of BSE [29]. Other studies across the various regions of Ghana have reported a higher proportion of women of reproductive age engaging in BSE [30, 31]. The variation in findings can be attributed to the fact that many of these alternative studies primarily focused on tertiary students, who have integrated breast cancer awareness and screening practices into their academic curriculum. Given the relatively low level of awareness and engagement in BSE among the study population, it is imperative to enhance awareness campaigns and educational programs focused on BSE within this demographic.

Marital status, religion, and family history of breast cancer did not demonstrate a significant association with knowledge about breast cancer risk factors and clinical presentation. However, employment status, age, and educational level were significantly associated with a better understanding of both risk factors and signs and symptoms of breast cancer. Most of the participants with sufficient knowledge of breast cancer risk factors and signs and symptoms had tertiary education compared to individuals with lower levels of education (*p* < 0.05). Previous studies elsewhere have also reported a significant association between education and knowledge of breast cancer risk factors as well as clinical presentation [24, 32]. Other studies indicate that women with higher levels of education have significantly better knowledge about breast cancer [33, 34]. Moreover, following awareness programs, there was a notable rise in breast cancer knowledge among college teachers across different states in India [35], which emphasizes the need for educational programs. The significant association observed between educational level and breast cancer knowledge may stem from increased exposure to and familiarity with breast cancer and related health topics through various courses, training sessions, and seminars. Individuals with higher levels of education often have greater access to information and resources, allowing them to stay informed about health issues including breast cancer. Therefore, it is imperative to implement initiatives aimed at broadening awareness regarding the symptoms and risk factors of breast cancer through health education campaigns facilitated by women‐friendly organizations.

The current study has several limitations that should be considered when interpreting the findings. The cross‐sectional design captures data at a single point in time, making it difficult to establish causal relationships between study variables. This study's comparatively small sample size may reduce statistical power and potentially restrict the ability to generalize findings to broader populations. The study was conducted at a single health facility, which indicates that the experiences observed may not fully reflect those in other settings, particularly in rural or underserved areas where healthcare dynamics may differ. Selection bias is also a possibility, as the recruitment process may have led to an uneven representation of certain subgroups, influencing the overall conclusions. Moreover, the reliability of the tools used for data collection, including self‐reported measures, may introduce measurement bias, which could affect the accuracy of the findings.

## 5 Conclusions

Despite high awareness of breast cancer, knowledge of its risk factors and symptoms was lacking, with over half unaware of BSE. Stakeholders should consider promoting community radio programs, training female community health volunteers, and integrating breast cancer education into postnatal care services to enhance women's knowledge of breast cancer, BSE, and available screening services.

## Author Contributions

Conceived and designed the study: R.O., W.S.A.J., R.A.B.B., and C.O. Enrolled patients: R.O., A.E., and P.A. Analyzed the data: A.E. and C.O. Wrote the original draft of the manuscript: All authors. Agree with manuscript results and conclusions: All authors.

## Ethics Statement

The study was reviewed and approved by the School of Anaesthesia Kumasi (MOH/SOAK/08‐24/96/27). Approval was also sought from the administration of the Bibiani Municipal Hospital prior to data collection. A written informed consent was obtained from all participants prior to questionnaire administration and participants were assured of anonymity and confidentiality.

## Conflicts of Interest

The authors declare no conflicts of interest.

## Data Availability

The data that support the findings of this study are available from the corresponding author upon reasonable request.

## References

[1] S. Lei , R. Zheng , S. Zhang , et al., “Global Patterns of Breast Cancer Incidence and Mortality: A Population‐Based Cancer Registry Data Analysis From 2000 to 2020,” Cancer Communications 41, no. 11 (2021): 1183–1194. 34399040 10.1002/cac2.12207 PMC8626596

[2] L. Lv , B. Zhao , J. Kang , S. Li , and H. Wu , “Trend of Disease Burden and Risk Factors of Breast Cancer in Developing Countries and Territories, From 1990 to 2019: Results From the Global Burden of Disease Study 2019,” Frontiers in Public Health 10 (2023): 1078191. 36726635 10.3389/fpubh.2022.1078191 PMC9884979

[3] P. Adewale Adeoye , “Epidemiology of Breast Cancer in Sub‐Saharan Africa,” in Breast Cancer Updates, ed. S. Sözen and S. Emir (IntechOpen, 2023).

[4] C. A. Anyigba , G. A. Awandare , and L. Paemka , “Breast Cancer in Sub‐Saharan Africa: The Current State and Uncertain Future,” Experimental Biology and Medicine 246, no. 12 (2021): 1377–1387. 33926257 10.1177/15353702211006047 PMC8243219

[5] S. Osei‐Afriyie , A. K. Addae , S. Oppong , H. Amu , E. Ampofo , and E. Osei , “Breast Cancer Awareness, Risk Factors and Screening Practices Among Future Health Professionals in Ghana: A Cross‐Sectional Study,” PLoS One 16, no. 6 (2021): e0253373. 34166407 10.1371/journal.pone.0253373 PMC8224936

[6] Y. A. Amoako , B. Awuah , R. Larsen‐Reindorf , et al., “Malignant Tumours in Urban Ghana: Evidence From the City of Kumasi,” BMC Cancer 19 (2019): 1–12. 30909876 10.1186/s12885-019-5480-0 PMC6434839

[7] S. Łukasiewicz , M. Czeczelewski , A. Forma , J. Baj , R. Sitarz , and A. Stanisławek , “Breast Cancer—Epidemiology, Risk Factors, Classification, Prognostic Markers, and Current Treatment Strategies—An Updated Review,” Cancers 13, no. 17 (2021): 4287. 34503097 10.3390/cancers13174287 PMC8428369

[8] V. Sopik , “International Variation in Breast Cancer Incidence and Mortality in Young Women,” Breast Cancer Research and Treatment 186 (2021): 497–507. 33145697 10.1007/s10549-020-06003-8

[9] Y. Liu , N. Nguyen , and G. A. Colditz , “Links Between Alcohol Consumption and Breast Cancer: A Look at the Evidence,” Women's Health 11, no. 1 (2015): 65–77. 10.2217/whe.14.62 PMC4299758 25581056

[10] J. L. Hopper , G. S. Dite , R. J. MacInnis , et al., “Age‐Specific Breast Cancer Risk by Body Mass Index and Familial Risk: Prospective Family Study Cohort (ProF‐SC),” Breast Cancer Research 20 (2018): 1–11. 30390716 10.1186/s13058-018-1056-1 PMC6215632

[11] M. E. Jones , M. J. Schoemaker , L. B. Wright , A. Ashworth , and A. J. Swerdlow , “Smoking and Risk of Breast Cancer in the Generations Study Cohort,” Breast Cancer Research 19 (2017): 1–14. 29162146 10.1186/s13058-017-0908-4 PMC5698948

[12] F. Z. Francies , R. Hull , R. Khanyile , and Z. Dlamini , “Breast Cancer in Low‐Middle Income Countries: Abnormality in Splicing and Lack of Targeted Treatment Options,” American Journal of Cancer Research 10, no. 5 (2020): 1568. 32509398 PMC7269781

[13] D. S. Al‐Mousa , M. Alakhras , S. Z. Hossain , et al., “Knowledge, Attitude and Practice Around Breast Cancer and Mammography Screening Among Jordanian Women,” Breast Cancer: Targets and Therapy 12 (2020): 231–242. 33204150 10.2147/BCTT.S275445 PMC7666976

[14] S. M. Albeshan , S. Z. Hossain , M. G. Mackey , and P. C. Brennan , “Can Breast Self‐Examination and Clinical Breast Examination Along With Increasing Breast Awareness Facilitate Earlier Detection of Breast Cancer in Populations With Advanced Stages at Diagnosis?,” Clinical Breast Cancer 20, no. 3 (2020): 194–200. 32147405 10.1016/j.clbc.2020.02.001

[15] H.‐M. Hsieh , W.‐C. Chang , C.‐T. Shen , Y. Liu , F.‐M. Chen , and Y.‐T. Kang , “Mediation Effect of Health Beliefs in the Relationship Between Health Knowledge and Uptake of Mammography in a National Breast Cancer Screening Program in Taiwan,” Journal of Cancer Education 36 (2021): 832–843. 32103458 10.1007/s13187-020-01711-7

[16] S. A. Rahman , A. Al–Marzouki , M. Otim , N. E. H. K. Khayat , R. Yousef , and P. Rahman , “Awareness About Breast Cancer and Breast Self‐Examination Among Female Students at the University of Sharjah: A Cross‐Sectional Study,” Asian Pacific Journal of Cancer Prevention 20, no. 6 (2019): 1901. 31244316 10.31557/APJCP.2019.20.6.1901 PMC7021607

[17] S. O. Azubuike , C. Muirhead , L. Hayes , and R. McNally , “Rising Global Burden of Breast Cancer: The Case of Sub‐Saharan Africa (With Emphasis on Nigeria) and Implications for Regional Development: A Review,” World Journal of Surgical Oncology 16, no. 1 (2018): 1–13. 29566711 10.1186/s12957-018-1345-2 PMC5863808

[18] M. Badakhsh , A. Balouchi , S. Taheri , S. Bouya , S. Ahmadidarehsima , and M. Aminifard , “Attitude and Practice Regarding Breast Cancer Early Detection Among Iranian Women: A Systematic Review,” Asian Pacific Journal of Cancer Prevention 19, no. 1 (2018): 9–16. 29373873 10.22034/APJCP.2018.19.1.9 PMC5844641

[19] E. Kudzawu , F. Agbokey , and C. S. Ahorlu , “A Cross Sectional Study of the Knowledge and Practice of Self‐Breast Examination Among Market Women at the Makola Shopping Mall, Accra, Ghana,” Advances in Breast Cancer Research 5, no. 3 (2016): 111–120.

[20] I. Shirazu , A.‐N. Mumuni , Y. B. Mensah , et al., “Awareness and Knowledge on Breast Cancer Screening Among Reproductive Aged Women in Some Parts of Ghana,” Health and Technology 14, no. 2 (2024): 317–327.

[21] C.‐B. Sama , B. Dzekem , J. Kehbila , et al., “Awareness of Breast Cancer and Breast Self‐Examination Among Female Undergraduate Students in a Higher Teachers Training College in Cameroon,” Pan African Medical Journal 28, no. 1 (2017): 164. 29255561 10.11604/pamj.2017.28.91.10986 PMC5724944

[22] M. Tomic , M.‐L. Vescan , and M.‐I. Ungureanu , “Exploring Female Medical Students' Knowledge, Attitudes, Practices, and Perceptions Related to Breast Cancer Screening: A Scoping Review,” Journal of Medicine and Life 16, no. 12 (2023): 1732–1739. 38585536 10.25122/jml-2023-0412 PMC10994607

[23] D. G. Moustafa , E. S. Abd‐Allah , and N. M. Taha , “Effect of a Breast‐Self Examination (BSE) Educational Intervention Among Female University Students,” American Journal of Nursing Science 4, no. 4 (2015): 159–165.

[24] B. M. Gebresillassie , E. A. Gebreyohannes , S. A. Belachew , and Y. K. Emiru , “Evaluation of Knowledge, Perception, and Risk Awareness About Breast Cancer and Its Treatment Outcome Among University of Gondar Students, Northwest Ethiopia,” Frontiers in Oncology 8 (2018): 501. 30456205 10.3389/fonc.2018.00501 PMC6230991

[25] D. O. Onwusah , M. U. Eboigbe , J. E. Arute , and A. A. Mgbahurike , “Knowledge and Awareness of Breast Cancer Among University Students in South‐South Nigeria,” Scholars Academic Journal of Pharmacy 6, no. 1 (2017): 4–15.

[26] R. Dadzi and A. Adam , “Assessment of Knowledge and Practice of Breast Self‐Examination Among Reproductive Age Women in Akatsi South District of Volta Region of Ghana,” PLoS One 14, no. 12 (2019): e0226925. 31887161 10.1371/journal.pone.0226925 PMC6936838

[27] F. Y. Fouelifack , R. P. Binyom , A. M. Ofeh , J. H. Fouedjio , and R. E. Mbu , “Knowledge, Attitude and Practice of Breast Self‐Examination Amongst Women in Two Communities of Cameroon,” Open Journal of Obstetrics and Gynecology 11, no. 6 (2021): 773–793.

[28] L. Conte , G. De Nunzio , R. Lupo , et al., “Breast Cancer Prevention: The Key Role of Population Screening, Breast Self‐Examination (BSE) and Technological Tools. Survey of Italian Women,” Journal of Cancer Education 38, no. 5 (2023): 1728–1742. 37400725 10.1007/s13187-023-02327-3 PMC10509132

[29] L. A. Fondjo , O. Owusu‐Afriyie , S. A. Sakyi , et al., “Comparative Assessment of Knowledge, Attitudes, and Practice of Breast Self‐Examination Among Female Secondary and Tertiary School Students in Ghana,” International Journal of Breast Cancer 2018 (2018): 7502047. 30151285 10.1155/2018/7502047 PMC6091363

[30] R. A. Amegbedzi , J. Komesuor , H. Amu , and E. E. Tarkang , “Factors Influencing the Practice of Breast Self‐Examination Among Female Tertiary Students in Ho Ghana,” Advances in Public Health 2022 (2022): 7724050.

[31] R. Nukpezah , R. Alenyorige , A.‐W. Inusah , et al., “Knowledge and Practice of Breast Self‐Examination Among Undergraduate Midwifery Students of the University for Development Studies, Ghana,” Journal of Scientific Research and Advances 22 (2021): 26–37.

[32] M. O. Abbas and M. Baig , “Knowledge and Practice Concerning Breast Cancer Risk Factors and Screening Among Females in UAE,” Asian Pacific Journal of Cancer Prevention 24, no. 2 (2023): 479–487. 36853296 10.31557/APJCP.2023.24.2.479 PMC10162630

[33] R. Sarker , M. S. Islam , M. S. Moonajilin , M. Rahman , H. A. Gesesew , and P. R. Ward , “Effectiveness of Educational Intervention on Breast Cancer Knowledge and Breast Self‐Examination Among Female University Students in Bangladesh: A Pre‐Post Quasi‐Experimental Study,” BMC Cancer 22, no. 1 (2022): 199. 35193526 10.1186/s12885-022-09311-y PMC8862195

[34] S. S. Abo Al‐Shiekh , M. A. Ibrahim , and Y. S. Alajerami , “Breast Cancer Knowledge and Practice of Breast Self‐Examination Among Female University Students, Gaza,” Scientific World Journal 2021, no. 1 (2021): 6640324. 34007246 10.1155/2021/6640324 PMC8100409

[35] A. D. Burke , J. W. Burns , S. Chakraborty , T. Saha , A. Ray , and D. M. Borsch , “Evaluation of Cancer Awareness, Cancer Education, and Prevention Intervention Techniques Among University‐Level Students in the United States and India,” Journal of Education Health Promotion 11, no. 1 (2022): 187. 36003241 10.4103/jehp.jehp_1422_21 PMC9393919

